# Cardiac progenitor cells application in cardiovascular disease

**DOI:** 10.15171/jcvtr.2017.22

**Published:** 2017-08-28

**Authors:** Hassan Amini, Jafar Rezaie, Armin Vosoughi, Reza Rahbarghazi, Mohammad Nouri

**Affiliations:** ^1^Stem Cell Research Center, Tabriz University of Medical Sciences, Tabriz, Iran; ^2^Department of Thoracic Surgery, Tabriz University of Medical Sciences, Tabriz, Iran; ^3^Neurosciences Research Center (NSRC), Tabriz University of Medical Sciences, Tabriz, Iran; ^4^Department of Applied Cell Sciences, Faculty of Advanced Medical Sciences, Tabriz University of Medical Sciences, Tabriz, Iran

**Keywords:** Cardiac Progenitor Cells, Cardiac Tissue, Regeneration

## Abstract

Stem cells (SCs) have special potency to differentiate into different types of cells, especially cardiomyocytes. In order to demonstrate the therapeutic applications of these cells, various investigations are recently being developed. Cardiac progenitor cells are endogenous cardiac SCs that found to express tyrosine kinase receptors, c-Kit and other stemness features in adult heart, contributing to the regeneration of cardiac tissue after injury. This lineage is able to efficiently trans-differentiate into different cell types such as cardiomyocytes, endothelial cells, and smooth muscle cells. Noticeably, several cardiac progenitor cells have been identified until yet. The therapeutic applications of cardiac SCs have been studied previously, which could introduce a novel therapeutic approach in the treatment of cardiac disorders. The current review enlightens the potency of cardiac progenitor cells features and differentiation capacity, with current applications in cardiovascular field.

## Introduction

Cardiovascular disease (CVD) could involve the cardiac tissue and circulatory system. Based on the data released by American Heart Association, CVD is thought to be the leading global cause of death accounts for more than 17.3 million deaths annually. By 2030, this number is thought to be reached to more than 23.6 million people. It has been well-established that death rate has declined to 38% in the last decade. Up to present, some therapeutic strategies have been developed to only to eliminate the clinical symptoms,^1^ but the application of novel alternative therapies for CVD remains to be answered. It is well known that CVD diseases *per se* could induce detrimental effects on cardiomyocytes via induction of cytotoxicity notably apoptosis, autophagy, and necrosis.^2^ Recently, regenerative medicine has introduced new avenue and a branch of science which facilitate the restoration of damaged tissues and organs in CVD example.^3^ Based on scientific opinion, stem cells (SCs) have potential to multipotentiality, self-renewability and giving rise to different lineages, and can potently differentiate into different functional cell types which makes them as suitable candidate for the regeneration of various organs, peculiarly cardiac tissue.^3^

The emergence of SCs and the possibility of their presence in various tissues contributed to identify cardiac SC or cardiac progenitor cells (CPCs). After the introduction of CPCs, the theory that cardiac cells are not able to regenerate cardiac tissue has been fade out and thereby many researchers began to determine the possibility of their experimental and clinical usage as a therapeutic agent.^4^ A plethora of experimental studies revealed that multipotent progenitor cells could be extensively administrated to be effective in reducing the infarcted areas of cardiac muscle.^5^ During tissue regeneration, CPCs are shifted from silent to active phase, contributing to their differentiation into new myocytes and endothelial cells.^6^ Despite the potency of these cells in healing, the cardiac regeneration is limited due to insufficient CPC number, formation of immature cardiomyocytes, the extent of infarcted area and low self-renewal potency of aged cases.^7^ In addition, many drawbacks have been reported for the CPCs and other SC types. Regarding adult SCs limitation in cardiac tissue, some authorities reported that SCs possess a low rate of proliferation, cardiomyogenic differentiation, unsuccessful cell engraftment into cardiac milieu with inconsistent outcomes either in clinical and preclinical studies. In some experiments, the administration of CPCs predisposed the risk of cardiac arrhythmias and teratoma formation.^8^ Despite these limitations, the global intention has been concentrated on the application of CPCs on different cardiac abnormalities, especially infarction. Based on released data, transplanted CPCs exerted their therapeutic effects through different mechanisms. They could potentially tans-differentiate into cardiac and endothelial lineages. The exogenous CPCs, in turn, activate endogenous resident progenitor cells. Modulations in the dynamics of extracellular matrix (ECM) and appropriate cardiomyocytes fusion are initiated after CPCs transplantation. CPCs have potency to release numerous factors via paracrine manner. For example, it was revealed that CPC-derived exosomes could augment anti-apoptotic, immune-modulatory or pro-angiogenic situation.^9^ In the current review article, we aimed to scrutinize and explore the common feature, differentiation capacity and restorative effects of CPCs based on current scientific literature.

### CPCs feature and differentiation capacity

CPCs are a mixed group of cells residing in different sites of the cardiac tissue such as epicardium, atria, and ventricles.^10^ First, Anverse and et al discovered the existence of CPCs.^11^ So far, many laboratories unveiled the striking heterogeneity in CPCs population.^12^ Through the past decade, researchers discovered CPCs population in human or murine embryonic and adult heart, showing variation on the expression of SC markers: c-Kit^positive^ CPCs; SC antigen-1 (Sca-1^positive^) CPCs; Islet-1 (Isl-1^positive^) CPCs and platelet-derived growth factor receptor-alpha (PDGFRα^+^) expressing CPCs are exemplified until now.^13^ In addition, cardiosphere-derived cells (CDCs); epicardium-derived cells; cardiac side population cells are other examples seen in literature.^14^ In embryonic development, the existence of CPCs in primarily first heart field accelerates the formation of myocytes of the linear heart tube. Sometimes, another population of undifferentiated CPCs functions next level of SC source in heart field supplying the existing tube with extra myocytes, endothelial and smooth muscle cells.^15^ These cell markers are thought to be indicated to specific roles in different period of developmental period. The exact explanation for the existence and responsibility of different CPC populations remain to be addressed.^16^

CPCs have the abilities of self-renewing and could differentiate into all three main cell types of cardiac tissue including, cardiomyocytes, smooth muscle cells and endothelial cells.^17^ During cardiac development, early cell lineage makes place into what is termed the first heart field (FHF) that forms the main part of the heart (left ventricle and other parts), and the second heart field (SHF) that forms part of right ventricle and atria and all of outflow tracts.^17^ Mesp1^+^ and FGF8 were found to be the earliest markers of the cardiac lineage in the gastrulating mesoderm. Even, some authorities discovered the existence of Mesp1^+^ cells only prior to gastrulation. These factors make progenitor cells to migrate to the lateral plate mesoderm where the population takes on a crescent shape which termed cardiac crescent.^18^

The expression of both Nkx2-5 and Isl-1 factors discriminates multipotent CPCs during cardiac development. These factors contribute to the activation of GATA4. GATA4 has an important role in CPCs development and in cardiac gene expression and along with SWI/SNF chromatin remodeling complex subunit facilitates cardiac gene induction. On the other hand, SWI/SNF chromatin remodeling complex subunit could mutually stimulate GATA factors to induce CPCs specification.^19^ Myocyte enhancer factor 2C (MEF2C) is conceived as another transcription factor which is critical for identification of early CPCs. Insulin-like growth factor-binding protein 5 (IGF-bp5), platelet-derived growth factor receptor alpha (PDGF-rα), and the transmembrane protein ODZ4 and VEGF receptor 2 (Flk-1/KDR) may also correlate with CPC feature.^20^

### Paracrine role of CPCs in the regeneration of injured cardiac tissue

It is well-established that CPCs could release numerous molecules to surrounding milieu in paracrine manner, resulting in the restoration of cardiac tissues after CVD and dilated cardiomyopathy.^21^ For instance, they can produce nano-scale packs, namely exosomes containing a typical number of molecules. Evidence indicated that exosomes are as key mediators of CDC-induced regeneration and even could be helpful cell-free therapeutic application of SCs.^22^ Additionally, the supportive role of CPCs on cardiac regeneration by enhancing angiogenesis and the reduction of the infarcted myocardium has previously proved.^23^ According to scientific beliefs, CPCs are repository of many factors, biological molecules and cytokines.^24^ In an experiment by Ibrahim and colleague, it was showed that CPC-derived exosomes tailored angiogenesis and stimulated cardiomyocyte survival and proliferation. They also showed exosomes improved CPCs therapeutic plasticity and notably the blockade of exosome secretion resulted in CPCs inefficiency.^22^ In another study, it was assumed CPC-derived exosomes promoted angiogenesis, neovascularization with concurrent cardiomyocyte proliferation, and apoptosis reduction.^25^ Further investigations revealed the existence of high amount of miR-146a in CPC derive exosomes which is able to heighten the therapeutic outcomes both in vitro and in vivo.^25^ Different type of paracrine factors have been directly secreted into CPCs supernatant. For examples, chemokines: thymus and activation-regulated chemokine-3, stromal cell derived factor-1 (SDF-1), 6C-kine; vascular growth factors: vascular endothelial growth factor (VEGF), erythropoietin, basic fibroblast growth factor (bFGF), osteopontin, SC factor (SCF) along with cardiac differentiation factors such as activin A, Dkk homolog-1, transforming growth factor beta have been documented. On the other hand, CPC-derived microvesicles encompass angiotensin-1; hepatocyte growth factor, bFGF, insulin-like growth factor 1, PDGF, SCF, SDF-1, and VEGF are able to exert cardio-protective properties by evoking neovascularization and cell recruitment into injured sites.^26^ Of note, CPC-conditioned medium has protective role on cardiomyocytes and tube formation of endothelial cells in vitro condition.^27^ For instance, administration of CPC-derived exosomes facilitated endothelial cell migration and diminished cardiomyocyte toxicity in a mouse model for acute infarction.^28^ It was shown the high amount of miR-21 and miR-451/144 resulted in significant cardioprotective effects by promoting cardiomyocyte survival in vivo.^29^ Intra-myocardial administration of CPC-associated exosomes into the infarcted sites reduced cardiomyocyte apoptosis and scar size in rat model by increasing the amount of survival cells, blood vessel density and preventing the destruction of ventricular function via reduced fibrosis.^30^

### The current status of SCs application in cardiovascular therapies

A study by Orlic et al in 2001 acclaimed that bone marrow-derived SCs could form successfully cardiomyocytes post-administration into an infarcted site in mouse model.^31^ As a result, numerous attempts have been made to ameliorate the transplantation efficiency, but it must be waited for successful and flawless improvement using different adult SCs.^32^ In contrast to bone marrow SCs, skeletal muscle SCs, pax-7, MyoD positive cells, showed a high degree of differentiation potency into contractile cardiomyocytes in in vitro condition, although they failed to form an appropriate gap junctions and successful fusion with adjacent cardiac cells. Considering the efficient differentiation into cardiomyocytes, one could not conclude that this SC type merit to produce physiologically normal cardiomyocytes.^33^ The discrepancy exists regarding cardiomyocyte differentiation of various adult SCs which could be related to method of administration, cell differentiation protocols, model of animals, age and etc.^34^ Similar to adult SCs, human embryonic SCs (hESCs) and induced pluripotent SCs (iPSCs) have been frequently reported to trans-differentiate into cardiac cells.^35^ Regarding the adverse effects and some limitations such as teratoma formation and incomplete cardiac differentiation, the translational and clinical trial phase of hESCs and iPSCs must be interpreted carefully.^36^ Therefore, a very limited number of clinical trials have been commenced to analyze the function of cardiomyocyte-derived from iPSCs.^37^ For instance, in a phase 1 clinical trial using iPSCs, different cardiac tissue, including sarcomeric proteins (cTnI, cTnT, sarcomeric, MHCs and actins), cardiac-specific transcription factors (Nkx2.5, Tbx5/20, Mesp1 GATA4, MEF2c and Isl1), gap junction proteins (Cx43), chamber-specific proteins (MLC2 V and MLC2A and ANP) and ion channel genes were obviously up-regulated.^8^

### Biological pacing ability of CPCs

**Figure 1:**
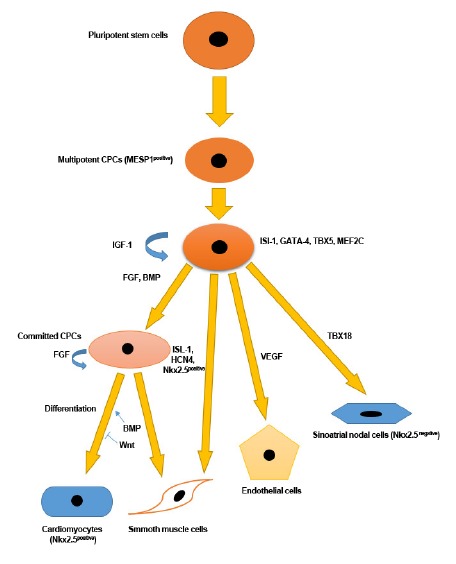
Different factors and signaling pathways participating in target differentiation of CPCs in developmental stage.

Some conditions can affect the pacemaker cells, either sino-atrial (SA) or atrioventricular nodes (AV), function, and normal physiological duties and thereby causing arrhythmia and malfunction in conducting of the electrical current through the cardiac tissue.^38^ Long-term use of electronic pacemakers could result in undesirable and some faults, leading possibly to devastating issues in the patients. Global RNA sequencing data revealed some essential transcription factors in pacemaker cells as seen in cardiomyocytes, notably Gata4, Mef2C, and Tbx5, but some are highly conserved and up-regulated pacemaker cells including Tbx18, Tbx3, Shox2, and Isl1 while some others are found in low level like Nkx2.5 (Figure 1).^39^

One method for generating AS node-like pacemaker cells (SANLPCs) is to modify and regulate signaling pathways of in the developmental stage of pluripotent SCs. Protze et al. previously described the generation of Nkx2.5^−/−^cardiomyocytes, functioning as pacemaker cells.^40^ The generated pacemaker cells had potential to express SA node cells markers, possessing pacemaker like action potential with the same ionic currents characteristics. During SANLPCs differential of SCs, bone morphogenic protein and retinoic acid signaling directed cells shift toward pacemaker phenotype. The inhibition of FGF pathways prevented the generation of Nkx2.5 positive cells. It has been reported that CPCs derived from embryonic heart tubes have the ability to efficiently trans-differentiate into pacemaker-like cells by engaging endothelin-1 factor and signaling.^41^ Recently, it was found tissue engineered cardiac pacemakers hold promising potential for ameliorating sinus node dysfunction. In Matrigel-coated surface, CPCs-derived pacemakers were juxtaposed with endothelial progenitor cells for the induction of sufficient vascularity and nourishment.^42^ The expression of Shox-2 induced embryonic SCs differentiation into pacemaker-like cells with an enhanced automaticity and prominent pacing capacity.^43^ In line with above-mentioned signaling pathways and factors, the potency of HCN4, a hallmark of pacemaker cells, must not be neglected during acquiring pacing functionality from different SCs and reprogramming protocols (Figure 1).^44^

Commensurate with these statements, the current approaches in order to generate biologic pacemakers are included either the production of functional pacemakers from embryonic pacemakers and expand pacemaker progenitors, then transplant these cells to the original position in the heart to integrate with resident cells, or accelerate differentiation of non-pacemaker cells into desired pacemaker cell phenotype.^45^

### The key role of CPC-derived exosomes on cardiac regeneration

Exosomes are nano-size particles secreted by vast arrays of mature and progenitor cells.^46^ Exosomes *per se* encompass a large number of biological materials related to lipidome, proteome, and transcriptome, contributing to direct cell to cell cross-talk.^47^ First, Gupta‏ and Knowlton reported rat cardiomyocytes (CMs) release intracellular exosomes to adjacent milieu while carrying a large amount of heat shock proteins (HSPs 70, 60, and 90) and other critical modulator of cell function.^48^ In this regard, authorities confirmed bio-functional role of CPC-and CMs-derived exosomes in various experimental conditions. For instance, CPCs-derived exosomes can provoke the migration and functional arrangement of endothelial cells (ECs).^28^ Based on the healthy and abnormal conditions, the content of exosomes could inspire different behavioral patterns to target cells. In a study conducted by Wang and colleagues, they declared CMs from diabetic rats distributed exosomes with potential to transfer miRNA-320. This microRNA initiated an anti-angiogenesis effect on adjacent ECs.^49^ Similar to CMs, CPCs exosomes prevent the occurrence of apoptosis in myocytes by transferring miR-21.^29^ Interestingly, the extent of CMs death and cardiac function was efficiently modulated after exposure to CPCs exosomes during myocardial infarction.^50^ These findings suggest interesting concept that exosomes from CPCs are key tools for the improvement of cardiac tissues and modulation of different cell function in various pathologies.

### Cardiovascular disease clinical trials based on the application of CPCs

According to statistics published on the PubMed database (https://clinicaltrials.gov/ct2/results?term=stem+cell+cardiac&Search), CPCs application is being increasingly for various heart conditions in human medicine. By May 2017, it was reported near to 21 CPC-based clinical trials have been successfully enrolled for patients (Table 1). A survey on the released data showed that the majority of clinical trials were related to myocardial infarction (~24%). Enthusiastically, most of these investigations confirmed the CPC-based benefits in the clinical treatment of patients who suffering from heart-related diseases.

**Table 1:** The list of CPC clinical trials recorded up to May 2017

**Condition/disease**	**Number of clinical trials**	**Clinical phase**
Myocardial infraction	5	I (2), II (2), IV (1)
Heart failure	3	II (2), ND
Ischemic cardiomyopathy	3	I (1), II (2)
Hypoplastic left heart syndrome	3	I (1), II (1), III (1)
Coronary artery disease	2	I (2)
Dilated cardiomyopathy	1	I (1)
Pulmonary arterial hypertension	1	I (1)
Heart transplantation	1	I, II (1)
Acute myocardial infraction	1	I, II (1)
Congestive heart failure	1	IV (1)

## Conclusion

Regarding the restorative potential of CPCs in trans-differentiationing into various cell types of cardiac tissue, CPCs pave a novel avenue for amelioration of different cardiovascular in human medicine. By applying in the field of CVD and efficient in vitro and ex vivo treatment make CPCs an appropriate candidate for restoration and regeneration of cardiac injuries in subjects.

## Ethical approval

Not applicable.

## Competing interests

The authors declare there is no conflict of interest**.**

## Acknowledgments

This review article is supported by a grant form Tabriz University of Medical Sciences (grant No. TBZMED.REC.1394.329).
